# Anterior and Posterior Meniscofemoral Ligaments: MRI Evaluation

**DOI:** 10.1155/2012/839724

**Published:** 2012-09-17

**Authors:** A. Bintoudi, K. Natsis, I. Tsitouridis

**Affiliations:** Radiology Department, Papageorgiou General Hospital “Papageorgiou”, 56403 Thessaloniki, Greece

## Abstract

Although meniscofemoral ligaments are distinct anatomic units, their anatomy and function are controversial from an anatomic and radiologic point of view. Five hundred knee MR examinations were retrospectively studied in an effort to demonstrate the incidence and variations regarding sex and age distribution, as well as the anatomy of the meniscofemoral ligament at magnetic resonance imaging. Patients were mostly men, three hundred and twelve, in contrast with women who were fewer, one hundred eighty-eight patients. The mean age of the patients who were included in this study was 46 years. More than half of them were between 20 and 40 years old; one hundred thirty-three patients among 20 to 30 years old and one hundred and one patients among 31 and 40 years old, in total two hundred thirty-four patients.

## 1. Introduction

An imaging breakthrough had led us to pay more attention in small anatomic structures such as the meniscofemoral ligaments. Meniscofemoral ligaments are straight bands of collagen that attach to the posterior horn of lateral meniscus and lateral part of medial femoral condyle [1]. For some authors, the meniscofemoral ligament is one ligament with two distinct bands, whereas for others are two distinct ligaments. The anterior meniscofemoral ligament (aMFL) which is leaning anterior to the posterior cruciate ligament (PCL) is also known as ligament of Humphrey, and the posterior meniscofemoral ligament (pMFL) leaning posterior to PCL is known as ligament of Wrisberg [1–6]. The incidence of the aMFL and pMFL ranges in the literature, although most of the studies are anatomic studies [2–7]. There are not many reports in the literature regarding magnetic imaging examination of the respective ligaments.The purpose of the present study is to elucidate the incidence of ligaments concerning the distribution among males and females and among patients with different ages.

## 2. Materials and Methods

Six hundred and three knee MRI examinations performed at our hospital during the period 2010-2011. Exclusion criteria include the patients with limitation on diagnosis due to motion artifacts and with imaging findings of PCL and lateral meniscus (LM) pathology. The remaining five hundred knee MRI exams were included in this retrospective study. The age of the patients ranged from 29 to 73 years (mean age 46 years). The patients were admitted for MRI exam either for chronic knee pain or after trauma.

All patients underwent MRI exams that were performed at 1 Tesla scanner (*Siemens Expert Plus)* using a phased-array knee coil. Each patient was positioned supine with the knee in a 10° flexion and 15° external rotation. The examination protocol included coronal and sagittal turbospin echo PD-WI and T2-WI, axial T2*-WI, and coronal STIR MR sequences, all with a slice thickness of 4 mm. No intravenous media contrast was administered. 

For the interpretation of MRI examination we paid special attention to coronal and sagittal PD-WI sequence and sagittal T2-WI sequence. The two ligaments, Humphrey and Wrisberg, were observed as a thin, linear band, with low MR signal intensity on coronal images anteriorly or posteriorly to PCL, respectively. On the sagittal images aMFL had a low MR signal, dot-like appearance located anterior to PCL and pMFL with the same appearance leaning posterior to PCL. 

The incidence of appearance, the different proportions in males and females, the MR sign and the occurrence were recorded. 

Ethical approval for this study was not obtained due to the fact that this is a retrospective study and was not needed.

## 3. Results 

**Figure 1:**
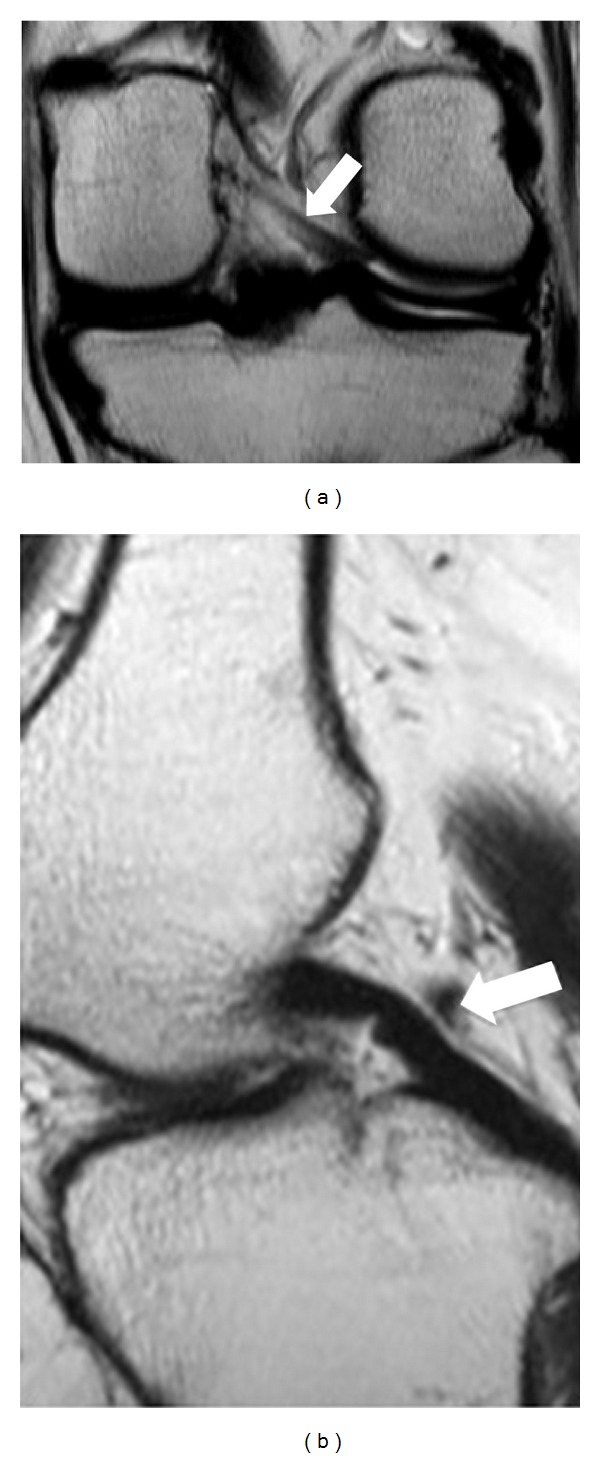
Coronal (a) PD-W image in which pMFL is demonstrating as a thick band and sagittal (b) PD-W image as a dot-like with low signal intensity posteriorly to posterior cruciate ligament (PCL) (white arrow).

From 603 knee MR examinations, 103 were excluded. The incidence of MFLs was evaluated in the remaining 500 knee MRIs. The pMFL or Wrisberg ligament was present in a very high percentage, 322 patients (64,4%), (Figure 1). Most of them in whom the pMFL was present were males, 240 patients (74,6%), and fewer, 82 patients (25,4%), were females. The visualization of the pMFL was easier and more frequently observed at the coronal sections (172/322/53%) rather than at the sagittal sections (150/322/47%). Although the incidence of appearance of Wrisberg ligament was high, it was usually thin and attached to PCL making the interpretation difficult. 

**Figure 2:**
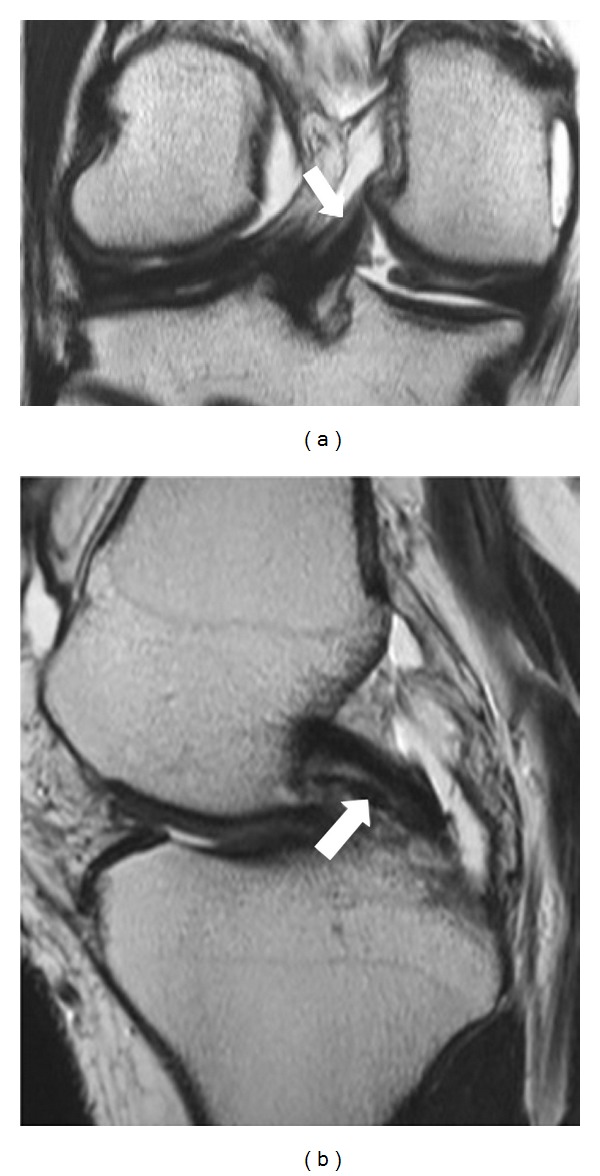
Coronal (a) PD-W image in which aMFL is depicted as a thick band and in sagittal (b) PD-W image as a dot-like with low signal intensity anteriorly to PCL (white arrow).

On the contrary, aMFL was present in a smaller number of patients, 59 patients (11,8%) (Figure 2). In this case the incidence of appearance in males was disproportional higher, 40 patients (67,8%), than in females, 19 patients (32,2%). Interpretation of the Humphrey ligament was easier at the sagittal images (34/59/57,6%) than in coronal (15/59/25,4%). 

**Figure 3:**
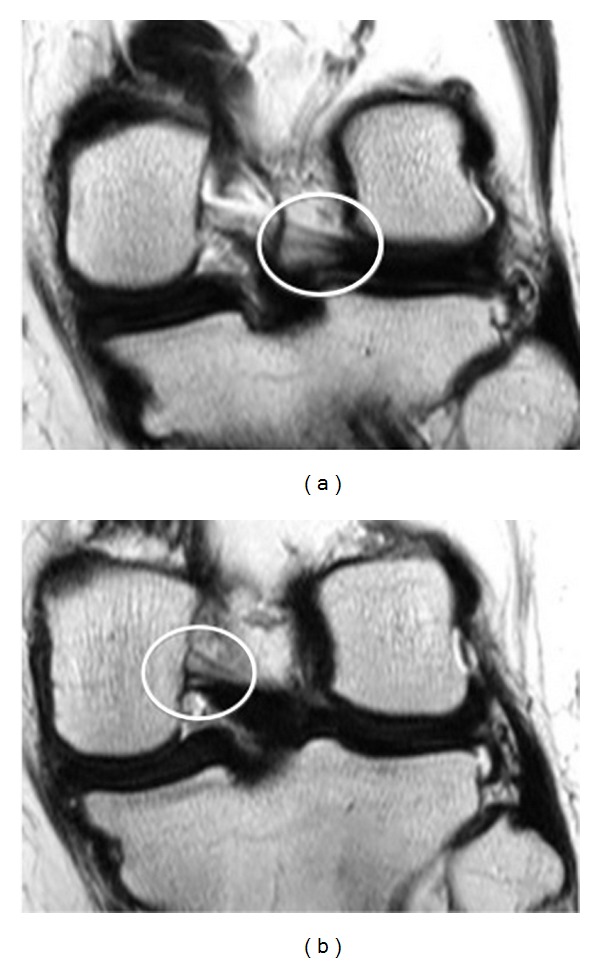
39 years old female who admitted to our hospital for chronic pain. Consecutive (a) (b) coronal PD-W image evaluate both aMFL and pMFL as thick bands with low signal intensity anteriorly and posteriorly, respectively, to PCL (white cycle).

**Figure 4:**
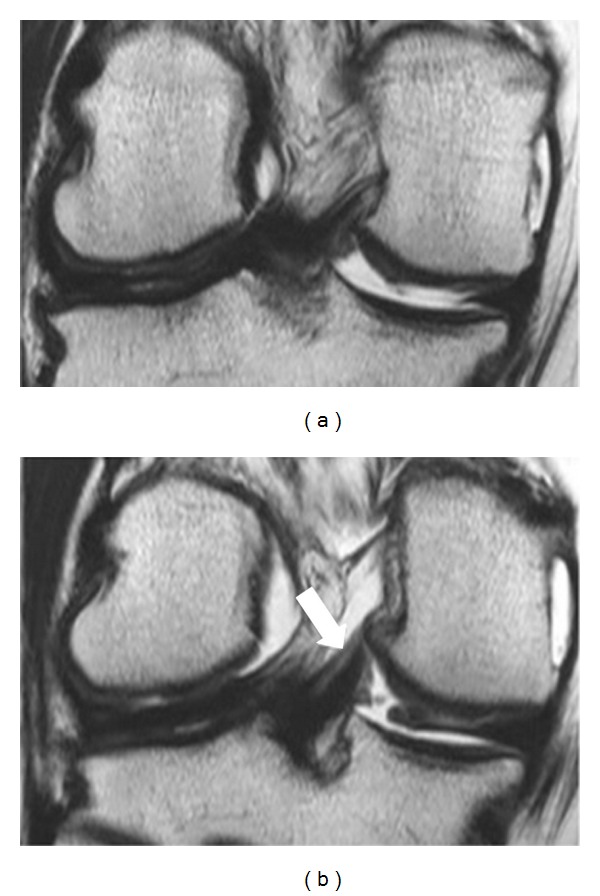
31 years old weekend football player, male, admitted to our hospital for medial meniscus tear. Consecutive (a) (b) coronal T2-W image demonstrating only the pMFL as a thick band with low signal intensity posteriorly to PCL. No fluid was present. MR examination was negative for meniscal tear (white arrow).

**Figure 5:**
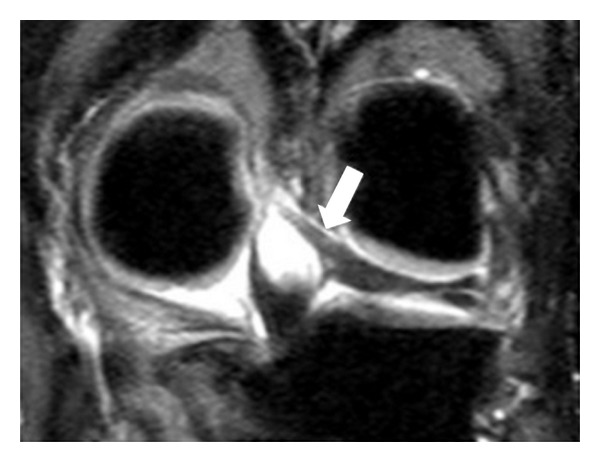
45 years old aerobic dancer, female, admitted to our hospital for trauma. Coronal STIR image demonstrating a very thick band with low signal intensity posteriorly to PCL, a large pMFL which plays the role of PCL (white arrow).

Both anterior and posterior meniscofemoral ligaments were present in 81 patients (37%) (Figure 3). Both ligaments were also more frequently observed in males, 44 patients (54,3%), than in females 37 (45,6%). The results are summarized in Table 1. Meniscofemoral ligaments were absent in 38 patients (7,6%). Finally, we separated our patients according to ages. Five different groups were formed. The first group included patients between 20 and 30 years old, the second 31 to 40 and go on until the last group in which patients older than 60 years old were included. First group consisted of 174 (34,8%) patients 98 (56,3%) males and 76 (43,6%) females, the second group 101 (20,2%) patients, 46 (45,5%) males (Figure 4) and 41 (44,5%) females, third group 111 (22,2%) patients, 69 (62,1%) males and 42 (37,8%) females, 59 patients between 50 and 60 years old 35 (71,1%) males and 24 (40,6%) females. Finally, the last group comprised of patients, 55 (11%) males and 20 (21%) females (Figure 5), respectively.  Table 2 summarizes the incidence of one or both ligaments and the number of patients with no ligament present with regard to age. 

The Wrisberg ligament was thicker than the Humphrey ligament. It was depicted with clarity at the coronal sections. On the other hand, Humphrey ligament was thinner and better visualized on sagittal images. 

## 4. Discussion

The anatomy, the function, and the imaging of the MFLs are a major issue among anatomists, orthopedics, and radiologists. The meniscofemoral ligaments connect the posterior horn of lateral meniscus with the lateral part of medial femoral condyle [1]. There are bands of collagen that attach firmly the posterior portion of the lateral meniscus during knee flexion [5, 8]. Poirier and Charpy first described it in 1892 [3]. The name of the third cruciate ligament was mistakenly used [9]. The name of ligament is also not correctly used because meniscofemoral ligament is not extended from a bone to another bone but from a fibrocartilage anatomic structure is the meniscus to a bone [9].

Embryological studies in human and animal knees proposed that MFL starts from posterior horn of lateral meniscus as a single band. The appearance of single or double MFL is due to the position of the PCL. Based on this evidence, different hypothesis was made for the variants which could present a meniscofemoral ligament [8]. Anatomically there have been described numerous variations of the scheme, proximal or distal insertions of the ligaments [6, 8, 10]. Anterior meniscofemoral ligament passes anterior to posterior cruciate ligament and there were described anatomic variants of the respective ligament. In the least frequent variant, the ligament consists of two or even three different bands with different origins from posterior horn of the lateral meniscus and different insertions at the femoral condyle. Most of the times variants are according to the size of the ligament, which could be small or large [8]. On the other hand posterior, meniscofemoral ligament, which passes posteriorly to posterior cruciate ligament, displays also anatomic variants.PMFL has described that could consist of two distinct bands having or not a hour-glass shape. Although is a thin ligament another anatomic variant describes a thick ligament, thicker than PCL. Of course all these variants are anatomically demonstrated and it might be difficult to observed them at knee MRI examinations [8].

 Anterior meniscofemoral ligament extents between the posterior portion of the posterior horn of the lateral meniscus and the femur, in the 10 o'clock position in a left knee, adjacent to the articular cartilage. Posterior meniscofemoral ligament leaning also between the anterior portion of the posterior horn on the lateral meniscus but at the femur it inserts at the medial part of the intercondylar notch near to insertion of the posteromedial band of the posterior cruciate ligament. This is the reason why fibers of the pMFL and PCL are sometimes intermingle [2, 8–11]. Meniscal insertion of the MFLs is possible to mimic the appearance of a tear. In our study, anatomic variations of MFLs were not evaluated. We try to describe specific details of MFLs, because as far as you concern, there have been very few studies with MRI at the respective issue.

 The function of the aMFL and the pMFL is not clearly understood. We know that MFLs play an important role as stabilizers and protectors for the posterolateral femorotibial compartment. They try during knee motion to increase congruity between the mobile lateral meniscus and lateral femoral condyle. They also play a protective role for the posterior horn of the lateral meniscus. The MFL has a totally different function during knee extension and flexion due to the tension, which is applied on pMFL and aMFL, which is totally different. They have reciprocal and non-isometric tensioning pattern. The aMFL is taught during flexion and lax during extension that is in contrast to the function of the pMFL. It is taut during extension and lax during flexion. The aMFL has a supplementary role to the anterior band of posterior cruciate ligament in contrast with the pMFL which supplements the function of the posterior band of the PCL [2–4, 10, 12–15]. Studies had shown that MFLs have a principal role during internal rotation of the tibia with a fixed foot [10]. There are authors who have implied that MFLs have functional similarities with posterior band of posterior cruciate ligament. For these reasons, most of the studies negotiated the antagonistic role of MFLs after partial or total tear of posterior cruciate ligament [4]. MFLs could act as a splint during injuries of the PCL giving the proper time to the ligament for conservative healing. It is important to be aware of the presence, anatomy, and specific difficulties and variations on MFLs. 

Imaging is adding important information regarding incidence of appearance. Several authors have shown, most through anatomic studies, the high prevalence of one of or both of MFLs. Anatomic studies, such as by Kusayama et al. and Amadi et al., demonstrate a very high incidence of 100%, thus other studies, such as Amis et al., a smaller incidence of 93% [2, 9, 15]. There is no radiological study with such a high incidence as it is show in the study by Amis et al. [2]. In recent radiological studies which were performed by Hassine et al., Gupte et al., Choi et al., Erbagci et al., and Lee et al., incidences range from 87% to 78% for the presence of at least one MFL [4–6, 14, 16]. In our study, the incidence of at least one MFL was almost 65%, which is in accordance with the other studies. The different incidence between anatomic and radiologic studies is due to or a partial volume averaging effect either to a slight difficulty on evaluation the ligament of Humphrey. 

Each meniscofemoral ligament was separately evaluated. The incidence of pMFL was higher than incidence of aMFL in all studies. Cho et al. visualized the ligament of Wrisberg in 84% of cases, Lee et al. 80%, and the smallest incidence was by Erbagci et al. 42% [5, 14, 16]. In our study pMFL was present in 322 patients (64,4%). The aMFL was present in a smaller incidence in all imaging studies. Cho et al., visualized the ligament of Humphrey in 15,8% of cases, Lee et al. 4%, and at the study of Erbagci et al. 12%. In our study aMFL was present in 59 patients (11,8%) [5, 14, 16]. Results in our research are smaller than in other studies maybe due to the large number of knee MRI examinations that were retrospectively studied.

We further divided the respective cohort regarding the sex of patients. The pMFL was present in 240 males, (74,6%) but fewer females, 82 patients (25,4%). Although Erbagci et al., visualized pMFL in a significant smaller cohort of 100 MRI knee examinations in 22 (52%) male patients and 20 (48%) females, the percentage of appearance is almost in accordance [15]. 

The aMFL was present in 40 males (67,8%), number disproportion higher than that of females 19 patients (32,2%). Amadi et al. visualized aMFL in 4 (33%) male patients and 8 (67%) females, which is in disagreement with our study [15]. Perhaps it is also due to the large number of knee MR images that are retrospectively studied.

Both MFLs were present at Moran et al. 28% and Lee et al. 1% [5, 13]. Erbagiet al. did not reveal any number [16]. In our study both MFLs were present in 81 patients (37%). Both MFLs were present in 44 males (54,3%) and 37 (45,6%) females. In the study by Erbagci et al., MFLs were present in 13 (46,4%) male patients and 15 (53,6%) females, which is in disagreement with our study [16]. Once more there is also a difference in findings at this group but is relative by smaller.

We observed that the Wrisberg ligament was thicker than Humphrey ligament. It was depicted with clarity at the coronal sections. On the other hand, Humphrey ligament was thinner and better visualized on sagittal images. Lee et al. reached the same conclusion [5]. 

In this study the incidence and appearance of meniscofemoral ligaments has been presented for different age groups. Gupte et al. and Cho et al. have proposed that the incidence is higher in younger patients, which is in totally agreement with our series [3, 4, 14]. 

The present study has limitations. The retrospective nature in the study design does not allow any arthroscopic or surgical correlations. 

## 5. Conclusions

The purpose of the present study was to give an overview of the radiologic prospective of the aMFL and pMFL. Degenerative cause might be able to explain the higher incidence in younger patients. The relatively large cohort of patients can contribute to the better knowledge of radiologic anatomy of meniscofemoral ligament and avert misdiagnosis of the aMFL and pMFL as loose bodies or PCL pathology.

## Figures and Tables

**Table 1:** Incidence of appearance of ligament of Wrisberg and ligament of Humphrey in male and female patients.

	aMFL	pMFL	aMFL + pMFL
Male	40	240	44
Female	19	82	37

**Table 2:** Incidence of appearance in different age groups.

Age group	aMFL	pMFL	aMFL + pMFL	Absent
20–30 y	17	132	24	1
31–40 y	13	57	29	2
41–50 y	16	77	16	2
51–60 y	8	33	8	10
>60 y	5	23	4	23
